# Ablation Resistance and Spray-Ability of Nano-Magnesium Silicate Reinforced Sprayable Silicone-Based Thermal Insulation Materials

**DOI:** 10.3390/nano16080476

**Published:** 2026-04-17

**Authors:** Junjie Hu, Yanbin Chen, Tingting Ge, Shuang Wu, Qianqiu Wu, Lifen Li, Yage Chen, Yifu Zhang, Yang Li

**Affiliations:** 1 National Key Laboratory of Aerospace Chemical Power, Xiangyang 441003, China; hujunjie@casc42.cn (J.H.); wushuang@casc42.cn (S.W.); wuqianqiu@casc42.cn (Q.W.); chenyage@casc42.cn (Y.C.); 2 Hubei Institute of Aerospace Chemotechnology, Xiangyang 441003, China; 3 College of Chemistry and Molecular Sciences, Wuhan University, Wuhan 430072, China; ht007226@whu.edu.cn (T.G.); ht007206@whu.edu.cn (L.L.); yfzhang2023@whu.edu.cn (Y.Z.)

**Keywords:** nanostructured magnesium silicate, silicone rubber, insulation materials, ablation resistance, spray-ability

## Abstract

In order to satisfy the requirement for lightweight, highly reliable sprayable silicone rubber insulation material (SASI) in next-generation spacecraft, and to achieve a synergistic balance among the sprayability, mechanical properties and ablation resistance of SASI, this paper describes the preparation of nanostructured magnesium silicate (n-MS) via a hydrothermal method and systematically investigates its effects on the sprayability, mechanical properties and ablation resistance of sprayable SASI. The findings suggest that when the n-MS loading is set at 15 parts, the linear ablation rate and mass ablation rate of the SASI under oxy-acetylene conditions are as low as 0.10 mm/s and 0.07 g/s, respectively, representing reductions of 41.8% and 67.1% compared to the unmodified samples. Building upon this enhancement in ablation resistance, the tensile strength was also increased by 3.70 MPa, representing a 19.3% increase. It is crucial to note that during the spraying process, the viscosity of the silicone rubber system remained within a narrow range of 540–550 mPa·s following the addition of this filler. This finding indicates that the introduction of n-MS had no significant adverse effect on the spraying process. In summary, n-MS has been demonstrated to enhance the mechanical strength and ablation resistance of silicone rubber materials while maintaining adequate spray coating performance. In comparison with conventional filled silicone rubbers, the sprayable silicone rubber insulating material developed in this study provides a new material basis for the future lightweight and intelligent development of aerospace engines.

## 1. Introduction

Thermal protection materials (TPS) for solid rocket motors are critical components located between the motor casing and propellant. They serve to block heat transfer and protect the casing from erosion by high-temperature and high-pressure gas generated during propellant combustion, thus playing an indispensable role in ensuring the stable operation of aerospace vehicles [1,2]. To meet the demands of lightweight construction and high reliability for new-generation aerospace vehicles, research on silicone rubber-based thermal insulation materials has shifted toward the development of products with low density, high mechanical performance, and excellent ablation resistance [3,4,5]. In addition, to reduce manufacturing complexity and improve the dimensional stability of insulation layers, preparation technology has gradually evolved from traditional manual laying to automated spraying [6], which imposes extremely strict requirements on the viscosity of insulation materials [7]. However, as a preferred insulation material for aerospace engines [8,9], silicone rubber still suffers from an inherent conflict between spray processability and comprehensive service performance, including mechanical properties and ablation resistance, making it difficult to match the current development trends of the industry [9].

In recent years, numerous nanofillers have been developed and employed as modifiers to improve and balance the mechanical and anti-ablation properties of silicone rubber insulation materials. Nanoparticulate silicates, with Si-O_4_ tetrahedra as their basic structural units, exhibit outstanding structural stability and high-temperature resistance. These materials have been increasingly investigated for the preparation of silicone rubber composites [10,11,12,13,14,15] and widely applied in aerospace thermal protection systems [16,17]. Typical examples include nanostructured magnesium silicate, aluminum silicate, and zirconium silicate [18,19]. Lu et al. [20] introduced magnesium silicate nanotubes as multifunctional fillers into silicone rubber. On one hand, the porous and hollow tubular structure of magnesium silicate enables mechanical interlocking with polymer chains inside the channels, effectively suppressing interfacial slippage and enhancing the mechanical properties of the composite. On the other hand, magnesium silicate catalyzes and modifies the thermal decomposition pathway of polymers at high temperatures, promoting the rapid formation of high-quality silicate-reinforced char. Such a stable char layer forms a physical barrier that inhibits mass and heat transfer, ultimately suppressing combustion and reducing ablation. Huang et al. [5,13] successfully synthesized HNTs@ZIF hybrid fillers with simultaneous reinforcing and flame-retardant effects. After incorporation into silicone rubber composites, the materials exhibited significantly improved flame retardancy and smoke suppression. When 2 wt.% HNTs@ZIF was added, the flame retardancy and smoke suppression of phosphorus-containing silicone rubber were further enhanced because cobalt ions in ZIF can catalyze the formation of char. The peak heat release rate (pHRR) and peak smoke production rate (pSPR) decreased by 18.3% and 16.3%, respectively, while the total heat release (THR) and total smoke production (TSP) dropped by 23.6% and 24.4%, respectively [21].

Given the exceptional anti-ablation characteristics and mechanical reinforcement potential of magnesium silicate, this study proposes an innovative strategy by introducing nanostructured magnesium silicate into a sprayable silicone rubber insulation system. The synergistic effect between nano-magnesium silicate and reinforcing fumed silica is systematically explored, aiming to enhance the mechanical and anti-ablation performance of sprayable silicone rubber insulation materials while maintaining high-quality spray processing. Accordingly, nanostructured magnesium silicate-reinforced sprayable silicone rubber insulation materials (SASI) were fabricated. Their spray feasibility was evaluated in terms of viscosity and spraying performance, and their comprehensive application properties were characterized through a series of tests, including mechanical properties (tensile strength, elongation at break, hardness, density), thermal stability, and ablation resistance. This innovative work is expected to provide a feasible strategy for designing next-generation ultra-high-temperature TPS with favorable spray-ability, high mechanical strength, and superior thermal performance.

## 2. Materials and Methods

### 2.1. Raw Materials and Experimental Equipment

Branched hydroxyl-terminated phenyl silicone oil (BHTPS), with a viscosity of 10,000 mPa·s, was self-prepared in the laboratory. Hydrophobic fumed silica (HFS), having a specific surface area of 200 m^2^/g, was purchased from Hubei Huifu Nanomaterial Co., Ltd., Yichang, China. Tetrapropoxysilane (TPOS) with a purity of 98% was obtained from Shanghai Macklin Biochemical Technology Co., Ltd., Shanghai, China. Modified carbon fiber (MCF), with a carbon fiber length of 0.3–0.5 mm, was self-prepared in the laboratory. Dibutyltin dilaurate (DBTDL) of industrial grade was purchased from Hubei Longsheng Xinsihai Technology Co., Ltd., Wuhan, China. Magnesium oxide, industrial grade with a purity of 98%, was supplied by Changsha Zhengya Chemical Co., Ltd., Changsha, China. Hollow glass microspheres, industrial grade with a bulk density of 0.32 g/cm^3^, were used as raw materials and purchased from Zhengzhou Shenglait Hollow Microsphere New Materials Co., Ltd., Zhengzhou, China. Petroleum ether (PE), with a boiling range of 90–120 °C, was purchased from Shanghai Macklin Biochemical Technology Co., Ltd.

### 2.2. Preparation of Nano-Magnesium Silicate

Magnesium oxide and silicon dioxide were weighed according to a molar ratio of 1:1. Magnesium oxide was uniformly dispersed in an organic/inorganic solvent, followed by the addition of silicon dioxide. The mixture was transferred into an autoclave for hydrothermal treatment at 120 °C for 10 h. After cooling to room temperature, a base was added to adjust the pH to 12, and the solution was stirred uniformly. Hydrothermal treatment was then continued at 210 °C for 120 h. After the reaction, the product was washed thoroughly with anhydrous ethanol and deionized water, centrifuged, and dried at 60 °C for further use. The as-prepared product was labeled as n-MS.

### 2.3. Preparation of Spray-Applied Silicone Rubber Thermal Insulation Materials

The sprayable silicone rubber insulation material was a condensation-type room-temperature vulcanized system. The detailed preparation procedure is as follows: 100 g of branched hydroxyl-terminated phenyl silicone oil, 20 g of hydrophobic fumed silica, and 40 g of modified carbon fiber filler were sequentially added into a dynamic mixer. The mixture was premixed at 300 r/min for 30 min, then heated to 120 °C and degassed under a negative pressure of 0.9 MPa for 2 h to remove moisture.

After cooling to ambient temperature, 8% crosslinking agent tetrapropoxysilane and 1.5% catalyst dibutyltin dilaurate were added, and mixing was continued for 10 min. A small amount of petroleum ether (boiling range 90–120 °C) was added as a diluent to adjust the viscosity to a sprayable level. The prepared compound was loaded into a spray tank and applied via airless spraying to form silicone rubber insulation samples with a thickness of 2 mm. The samples were cured at room temperature for 7 days.

The content of HFS/n-MS was set as a variable. The sample without n-MS was labeled SASI-20/0. To investigate the effect of n-MS, 5 g, 10 g, 15 g, and 20 g of n-MS were incorporated, corresponding to SASI-20/5, SASI-20/10, SASI-20/15, and SASI-20/20, respectively. To explore the synergistic effect between n-MS and HFS, the dosage of HFS was also varied, giving SASI-15/20, SASI-10/20, and SASI-5/20. The detailed formulations are listed in Table 1.

### 2.4. Characterization

To clarify the microstructure morphology, thermal stability, and decomposition behavior of nano-magnesium silicate, this study integrated multiple characterization techniques to conduct a systematic analysis of its physical and chemical structures, involving four core methods: scanning electron microscopy (SEM), powder X-ray diffraction (XRD), Fourier-transform infrared spectroscopy (FTIR), and thermogravimetric analysis (TGA). Specifically, scanning electron microscopy (SEM) measurements were conducted under a high-vacuum environment with a vacuum degree of 1.0 × 10^−5^ Pa. Prior to measurement, all samples were sprayed with a thin layer of gold (spraying thickness: 5–10 nm) using an ion sputter coater. The SEM observations were carried out at an acceleration voltage of 15 kV to obtain high-resolution microstructural information of the samples. Powder XRD measurements were carried out on a Shimadzu XRD-6100 diffractometer (Japan), with copper Kα radiation employed as the test light source, the radiation wavelength set to 0.1542 nm, the scanning range adjusted from 10° to 80°, and the scanning rate stabilized at 10°·min^−1^. Thermogravimetric analysis was implemented on a Netzsch STA-2500 simultaneous thermal analyzer under an air atmosphere, with a heating rate maintained at 10 °C·min^−1^ and a temperature range covering 30~800 °C. Unless otherwise specified, all subsequent spray-cured silicone rubber thermal insulation material samples were subjected to various performance characterizations under the same test conditions to ensure the comparability of the test results.

Construction processability is one of the core evaluation indicators for spray-type silicone rubber thermal insulation samples. Therefore, this study focused on the viscosity characterization of the diluted solution of the prepared spray-type silicone rubber thermal insulation samples. The viscosity tests were performed using a DNJ-79 digital viscometer equipped with a No. 3 rotor; the shear rate was set to 0.6 r/min, and the test temperature was kept constant at 25 °C to ensure the accuracy of the viscosity data.

The rheological properties of the samples were characterized using a Malvern MCR 302 rotational rheometer equipped with a parallel-plate fixture (diameter 20 mm, gap 0.5 mm). All tests were performed in steady-state flow mode at a strictly controlled temperature of 25 ± 0.1 °C, and the shear rate sweep range was set from 0.01 to 1000 s^−1^.

The shear-thinning ratio was calculated from the low-shear viscosity at $\dot{\gamma }$ = 0.05 s^−1^ and the high-shear viscosity at $\dot{\gamma }$ = 500 s^−1^. The shear stress–shear rate data in the low-shear region ($\dot{\gamma }$ < 1 s^−1^) were fitted using the Casson model, whose expression is as follows, corresponding to Equation (1):

(1) $$\sqrt{\tau }=\sqrt{\tau_{0}}+\sqrt{\eta_{ca}\cdot \dot{\gamma }}$$

In Equation (1), *τ* is the shear stress (Pa); *τ*_0_ is the Casson yield stress (Pa), representing the strength of the internal network structure of the system; *η_ca_* is the Casson viscosity (Pa·s); $\dot{\gamma }$ is the shear rate (s^−1^).

The goodness of fit R^2^ was used to evaluate the fitting quality.

For the spray-cured silicone rubber thermal insulation samples, their tensile strength and elongation at break were tested using a UTM6503 computer-controlled electronic universal testing machine. Samples were prepared strictly in accordance with the GB/T 528 standard [22] and cut into standard dumbbell-shaped specimens (Type II, 75 mm × 4 mm) using a standard mold, with the tensile test rate set to 500 mm/min. Meanwhile, the hardness and density of the spray-cured samples were tested using an SHA-III Shore hardness tester and an SJ-600GY solid–liquid integrated density meter (manufactured by Shanghai Shuju Scientific Instruments Co., Ltd.), respectively. Both tests were conducted under a constant temperature environment of 25 °C to reduce the interference of temperature on the test results.

To comprehensively evaluate the ablation resistance of the spray-cured silicone rubber thermal insulation samples, the tested materials were processed into cylindrical specimens with a diameter of 30 mm and a height of 50 mm in accordance with the GJB 323A standard [23]. Before the test, the initial mass (m_1_) and initial height (h_1_) of each specimen were accurately measured. During the ablation resistance test, the flame temperature was precisely controlled at 3100 ± 100 °C, the spray gun was kept perpendicular to the specimen surface, and the distance between the nozzle and the specimen surface was fixed at 10 mm. Meanwhile, the oxygen flow rate and acetylene flow rate were reasonably set to 12 L/min and 8 L/min, respectively, to ensure the stability of the ablation conditions. During the test, the specimen was first fixed on a high-temperature-resistant fixture, and the spray gun was ignited to continuously ablate the center of the specimen for 20 s. After ablation, the specimen was naturally cooled to room temperature, and then its residual mass (m_2_) and residual height (h_2_) were accurately measured to provide basic data for the subsequent calculation of ablation rates.

The specific calculation methods for the linear ablation rate and mass ablation rate are as follows, corresponding to Equation (2) and Equation (3), respectively:

(2) $$R_{l}=\frac{h_{1}-h_{2}}{t}$$

In Equation (2), $R_{l}\;$ denotes the linear ablation rate, $h_{1}\;$ is the initial height of the specimen, $h_{2}$ is the residual height of the specimen after ablation, and *t* is the ablation time.

(3) $$R_{m}=\frac{m_{1}-m_{2}}{t}$$

In Equation (3), $R_{m}$ denotes the mass ablation rate, $m_{1}$ is the initial mass of the specimen, $m_{2}$ is the residual mass of the specimen after ablation, and t  is the ablation time.

## 3. Results and Discussion

### 3.1. Structural Characterization of the Synthesized n-MS

This study employs a structure–function integration design philosophy to enhance the ablation resistance of SASI materials by combining nanostructured magnesium silicate with a unique morphology with sprayable, room-temperature vulcanising silicone rubber. This results in a sprayable silicone rubber thermal insulation layer that combines excellent mechanical strength with ablation resistance. Magnesium silicate (n-MS) with a nano-florette morphology was synthesized via a controlled hydrothermal process by controlling the concentrations of magnesium oxide and silicon dioxide reactants. The material’s chemical composition and microstructure were analyzed using various characterization techniques to establish the relationship between its structure and reinforcing and ablation-resistant properties.

**Figure 1:**
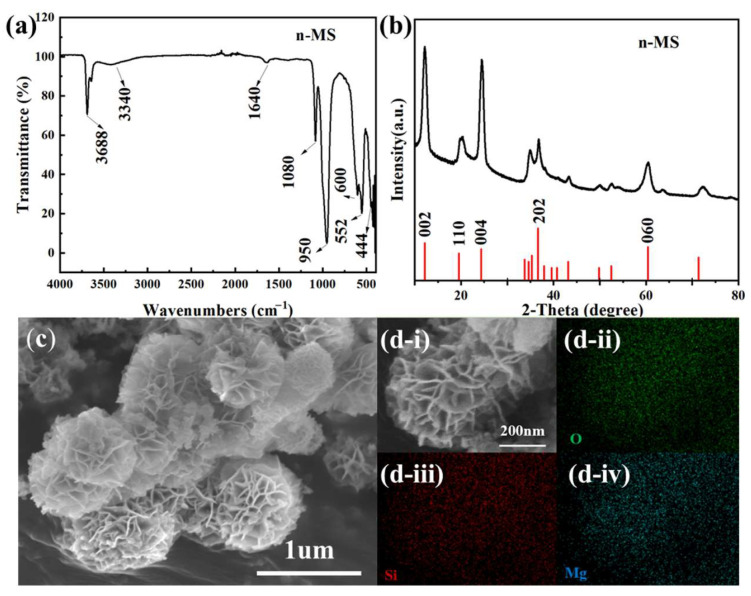
(**a**) FTIR of n-MS; (**b**) XRD pattern of n-MS; (**c**) SEM image of n-MS and (**d**) Elemental mapping of n-MS.

First, the bonding structure of the hydrothermal product was characterized by Fourier-transform infrared (FT-IR) spectroscopy. Figure 1a presents the FT-IR spectrum of n-MS. As shown in Figure 1a, characteristic absorption peaks of the hydrothermal product appear at 3688 cm^−1^, 3440 cm^−1^, 1640 cm^−1^, 1081 cm^−1^, 951 cm^−1^, 600 cm^−1^, 552 cm^−1^, and 444 cm^−1^. The peaks at 3688 cm^−1^ and 3440 cm^−1^ correspond to the stretching and bending vibrations of -OH groups, respectively. The bands near 1081 cm^−1^ and 951 cm^−1^ are attributed to the stretching and bending vibrations of Si-O bonds. The peaks around 600 cm^−1^ and 444 cm^−1^ belong to the perpendicular stretching and bending vibration modes of Si-O-Mg groups, respectively, which are consistent with those reported in the literature [24]. These results confirm that the hydrothermal product is a magnesium silicate compound.

X-ray diffraction (XRD) was employed to analyze the crystal structure of the synthesized product, and the corresponding XRD pattern is displayed in Figure 1b. As illustrated in Figure 1b, diffraction peaks of the hydrothermal product are observed at 2θ = 11.8°, 19.1°, 24.3°, 31.5°, and 60.9°, which can be assigned to the (002), (110), (004), (202), and (060) crystal planes, respectively. These signals are in good agreement with the characteristic peaks of the standard card (PDF#52-1562), further verifying that the product obtained after hydrothermal treatment is pure Mg_3_Si_2_O_5_(OH)_4_ (PDF#52-1562).

Figure 1c,d show SEM images of the hydrothermal magnesium silicate product. It can be clearly observed from Figure 1c,d that the synthesized magnesium silicate exhibits a uniform nanoflower-like structure with a diameter of approximately 500 nm. It can be inferred that Mg(OH)_2_ derived from the hydrolysis of MgO reacts with dissolved SiO_2_ to form magnesium silicate nanoparticles, which assemble into a nanoflower morphology.

**Figure 2:**
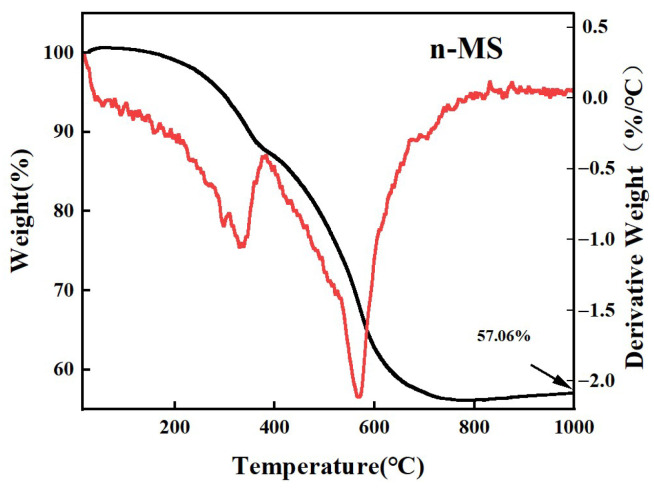
TG curves of n-MS.

In order to investigate the thermal stability of the designed nanofloral magnesium silicate (n-MS) and its structural changes during the ablation of silicone rubber, thermal gravimetric analysis (TGA) was employed to characterize its thermal behavior; the results of which are shown in Figure 2. The overall weight loss trend is consistent with the TGA results for magnesium silicate reported in the literature [25]. From the perspective of material structure design, the thermal weight loss process comprises two stages. Firstly, from room temperature to 380.8 °C, there is a removal of physically adsorbed water from the n-MS nanoflorette structure. Secondly, between 380.8 °C and 640.2 °C, there is a dehydroxylation reaction of hydroxyl groups coordinated to magnesium and silicon. It is noteworthy that a minor, broad endothermic peak emerges at 586 °C, thereby further substantiating the thermally induced elimination of structural hydroxyl groups within the octahedral layered structure of n-MS. At this temperature, accompanied by the loss of structural water, n-MS begins to undergo a phase transition [26], with its layered structure gradually evolving into a more stable phase. This thermal behavior provides significant evidence for the structural integrity of n-MS under high-temperature conditions and its application as a functional ablation-resistant filler.

### 3.2. Investigation on the Sprayability of n-MS in the SASI

The utilization of spray coating technology confers a number of substantial advantages in the domain of preparing diverse functional material layers. These advantages encompass the technology’s ease of operation, its high film-forming efficiency, its controllable film thickness (less than 1 mm), its uniform coatings with few defects, and its cost-effectiveness [27,28]. In the context of silicone rubber insulating materials, the efficacy of the spray coating process is twofold: firstly, it enhances molding efficiency, and secondly, it reduces defects introduced during preparation by traditional molding methods. However, the efficacy of spray coating is contingent on the rheological behavior of the material; the incorporation of fillers frequently results in substantial alterations to the system’s viscosity, consequently impacting the quality of the coating. Consequently, the key issue that must be addressed in material design is how to maintain good spray coating process adaptability whilst enhancing the material’s ablation resistance and mechanical properties.

**Figure 3:**
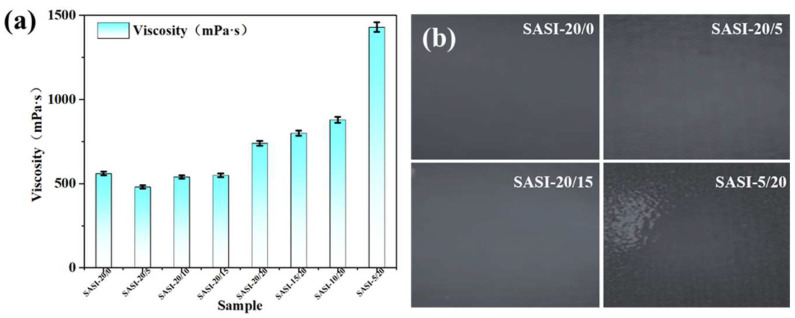
(**a**) Viscosity of SASI sprayable materials with different n-MS addition amounts; (**b**) Spraying effects of SASI materials with 4 different n-MS contents.

In consideration of the aforementioned factors, the present study systematically investigated the impact of introducing nano-magnetite (n-MS) as a functional filler into sprayable silicone rubber insulation (SASI) on the processing properties of the composite material. Initially, the viscosity of the SASI spray coating was examined at varying n-MS loading levels, with the outcomes presented in Figure 3a. Subsequently, the mixture was loaded into a spray booth and sprayed under conditions of 0.3 MPa atomisation pressure and a spray distance of approximately 30 cm, with a 2 min interval between each spray pass and a spray gun travel speed of 300 mm/s, resulting in a final coating thickness of 2–3 mm. Figure 3b illustrates the surface morphology of the coatings obtained after three spraying passes for four groups with different n-MS contents. This assessment was used to evaluate the effect of filler content on the coating’s forming quality.

In order to systematically investigate the effect of incorporating nano-magnetite (n-MS) into the material on its processability, this study analyzed the rheological behavior of the SASI composite system at different n-MS loading levels. The results are displayed in Figure 3a. As the n-MS content increased, the viscosity of the SASI spray coating exhibited a non-monotonic trend, initially decreasing and then increasing. In particular, the viscosity of the SASI-20/5, SASI-20/10 and SASI-20/15 samples was lower than that of the control system without n-MS, demonstrating good spray flowability. An increase in the n-MS content to 20 parts (i.e., SASI-20/20) resulted in a marked increase in the system viscosity. Moreover, as the HFS content diminished, the system viscosity persisted in its upward trajectory. From the perspective of interfacial design, this phenomenon can be attributed to the lower hydroxyl content on the surface of the modified HFS. Consequently, interactions with the main silicone rubber chains are limited to van der Waals forces, which is relatively weak, resulting in low resistance to interchain motion, where as a minimal quantity of hydroxyl groups persists on the surface of n-MS [29]. These polar hydroxyl groups engage with the silicone rubber molecular chains through the process of hydrogen bonding. Concurrently, a three-dimensional hydrogen-bonded network structure formed by n-MS and siloxane molecular chains may exist, which significantly restricts the movement of the molecular chains. When considered in conjunction with synergistic effects such as the adsorption and entanglement of molecular chains on the particle surface, as well as the restriction of free volume, this results in the system exhibiting significant shear-thinning thixotropic behavior. It has been established that, upon the attainment of a critical value of hydroxyl density on the filler surface, the network structure is strengthened, the viscosity of the system increases significantly, and the uniformity of filler dispersion consequently decreases. As previously stated, the quality of the spray-molding process is closely related to the viscosity of the system. As demonstrated in Figure 3b, when the viscosity of the SASI system is regulated within 1000 mPa·s, the resultant coating surface is characterized by smoothness, continuity and uniformity, thereby ensuring optimal outcomes in the context of spray-molding processes. In contrast, the viscosity of the SASI-5/20 sample attained a maximum of 1430 mPa·s, giving rise to orange peel and agglomeration defects on the coating surface. In consideration of the established correlation between the aforementioned rheological behavior and forming quality, the present study imposed a limitation on the design loading of n-MS, confining it to a maximum of 15 parts, with the objective of attaining an optimal balance between material reinforcement and the compatibility of the spray coating process.

**Figure 4:**
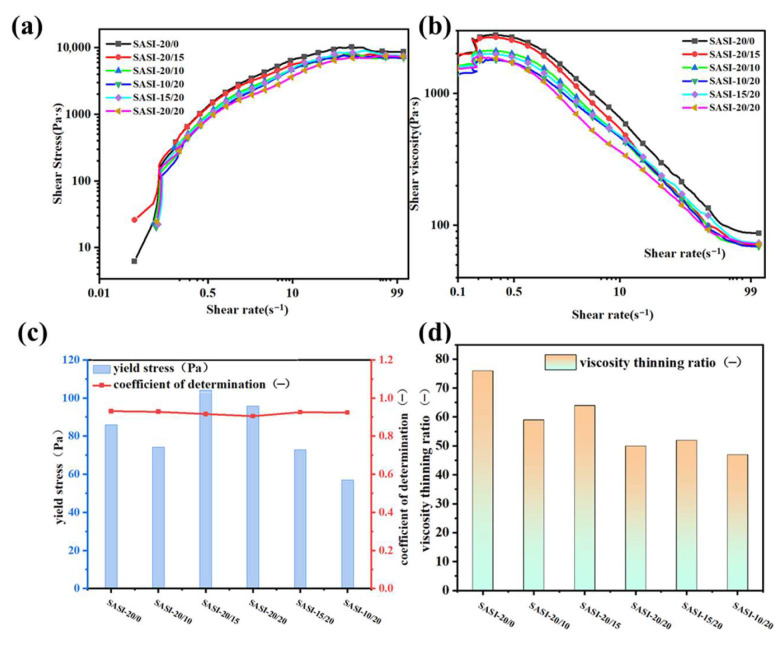
Steady-state flow curves of silicone rubber coating samples with different composite addition amounts of fumed silica/magnesium silicate. (**a**) shows the double-logarithmic plot of shear stress versus shear rate, (**b**) presents the double-logarithmic plot of viscosity versus shear rate, (**c**) displays the yield stress in the low-shear region and the fitting degree after fitting by the Casson model, and (**d**) illustrates the shear viscosity thinning ratio of each formulation.

As shown in Figure 4a, the shear stress of all samples increases monotonically with shear rate, displaying a good linear relationship in the double-logarithmic coordinate system, which is characteristic of pseudoplastic fluids and consistent with the ideal rheological properties of silicone rubber coatings. SASI-20/0 exhibits the lowest shear stress, while SASI-20/15 shows the highest [30].

Figure 4b demonstrates that the apparent viscosity of all samples decreases significantly with increasing shear rate, showing strong shear-thinning behavior—critical for efficient spraying: high viscosity at low shear prevents filler sedimentation and coating sagging, while reduced viscosity at high shear facilitates atomization and spreading. At high-shear rates, the apparent viscosity stabilizes at 15.1–19.2 Pa·s with small variations, indicating that the composite filler content has a limited impact on high-shear fluidity, and all samples meet spraying requirements.

Yield stress, dependent on the internal network strength of the system and regulated by filler dispersion and synergistic effects, tends to first increase and then decrease with increasing magnesium silicate content (Figure 4c). Below 15 phr, uniform dispersion of fumed silica and magnesium silicate increases interparticle contact points and enhances interactions (e.g., hydrogen bonds, van der Waals forces), helping to perfect the synergistically formed three-dimensional network and thus significantly increasing yield stress. Above 15 phr, filler agglomeration may disrupt the network structure, leading to a reduction in yield stress.

Figure 4d shows that the shear-thinning ratio generally first increases and then decreases with rising magnesium silicate content. SASI-20/0 exhibits the highest ratio, which can be attributed to the uniform dispersion of pure fumed silica that forms a relatively regular shear-sensitive network. When magnesium silicate reaches 15 phr, the ratio decreases to 64, possibly due to filler agglomeration that impairs the network structure and weakens the synergistic effects between fillers.

### 3.3. Investigation on the Application Properties of n-MS in the SASI

**Figure 5:**
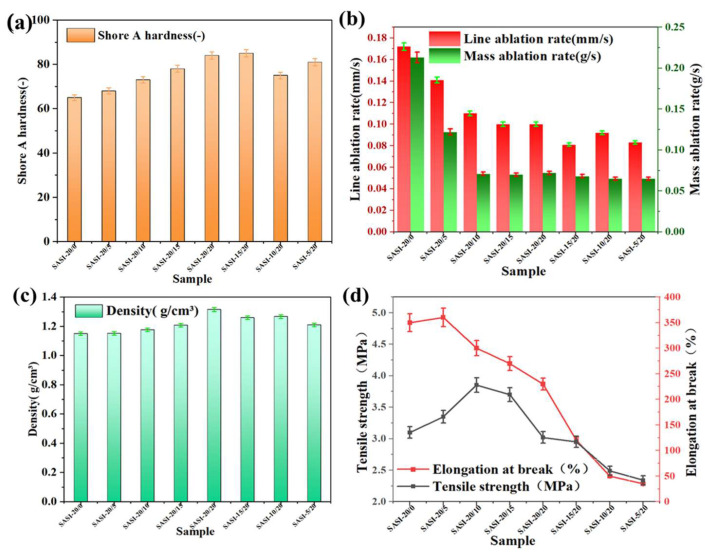
Characterization of application properties of SASI materials with different n-MS addition amounts: (**a**) Shore A hardness; (**b**) linear ablation rate and mass ablation rate; (**c**) density; (**d**) tensile strength and elongation at break.

The safety and reliability of aerospace vehicles during operation impose stringent requirements on the performance of silicone rubber insulation materials, which mainly include key parameters such as density, hardness, tensile strength, elongation at break, and linear ablation rate. To explore the regulatory effect of n-MS on the application properties of the SASI system, this study comprehensively characterized the material properties using the aforementioned key indicators. The characterization results of the SASI system with different n-MS addition amounts are presented in Figure 5.

Hardness serves as a pivotal indicator for evaluating the overall cross-linking density of the material as well as the interfacial interaction between fillers and the matrix. As presented in Figure 5a, with the total filler content maintained unchanged, the Shore A hardness of the SASI samples exhibits a gradual upward trend as the n-MS loading increases, and a maximum hardness of 85 is achieved in the SASI-15/20 specimen. This variation can be largely attributed to the unique nanoflower-like morphology of n-MS, which possesses a relatively high specific surface area and surface activity. Such structural features are favorable for strengthening interfacial adhesion with the silicone rubber matrix and effectively restricting the movement of molecular chains, thus leading to an obvious enhancement in the rigidity of the final material.

Abrasion resistance is a pivotal metric for evaluating the thermal protection capabilities of SASI materials. As demonstrated in Figure 5b, both the linear abrasion rate and the mass abrasion rate of the specimens decreased significantly with increasing n-MS content, indicating that n-MS plays a positive role in enhancing the material’s abrasion resistance. From a materials design perspective, this enhancement can be attributed to the stable ceramicised structure formed by n-MS at high temperatures, which effectively hinders the transfer of heat into the material’s interior. In addition, the phase transformation process of the material at elevated temperatures absorbs a proportion of the heat, thereby also contributing positively to the enhancement of ablation resistance. In summary, the incorporation of n-MS can effectively absorb more thermal energy, which in turn slows down the rate of temperature rise in the material. Meanwhile, it tends to enhance the structural strength of the material after ablation, rendering the material less likely to be eroded by high-speed gas flows and particles. Collectively, these effects contribute to a relatively superior thermal resistance performance of the material.

Changes in density are indicative of the equilibrium between the intrinsic properties of the filler and the overall quality of the material [31,32]. As demonstrated in Figure 5c, the SASI-20/20 specimen demonstrates the maximum density, at 1.315 g/cm^3^. Maintaining the total filler content constant, it is evident that an increase in the proportion of n-MS results in a corresponding rise in sample density. This phenomenon can be attributed primarily to the significantly higher density of n-MS (3.19–3.21 g/cm^3^) in comparison to HFS (2.0–2.2 g/cm^3^). It is noteworthy that despite the high intrinsic density of n-MS, the overall increase in material density is constrained due to the low packing density enabled by its nanoflor-like structure, thereby offering potential for lightweight design.

The mechanical properties manifest a non-monotonic variation, which is closely related to the composition of the filler, as illustrated in Figure 5d. Maintaining the total filler content constant, it was demonstrated that specimens exhibiting a higher proportion of n-MS demonstrated a significant decrease in both tensile strength and elongation at break. This finding indicates that, at higher loading levels, the direct reinforcing effect of n-MS on the matrix is weaker than that of HFS. From a materials design perspective, a higher n-MS content tends to result in a corresponding rise in the viscosity of the system, which may cause the nanoflower structures to become susceptible to agglomeration and the formation of stress concentration points. Such defects can in turn promote premature fracture under tensile loading [33,34].

However, for specimens with a fixed HFS content, when n-MS was incorporated at suitable levels, the tensile strength and elongation at break were observed to first increase and then decrease. This observation suggests that, within an appropriate loading window, n-MS is still capable of exerting a certain reinforcing effect on the silicone rubber matrix by means of favorable dispersion and enhanced interfacial bonding.

This study built upon the aforementioned analysis of macroscopic properties. It further characterized the thermal decomposition behavior of SASI composites using thermogravimetric analysis (TG). By observing the microstructure of specimens following oxy-acetylene ablation, the intrinsic mechanism by which n-MS regulates the flame-retardant and ablation-resistant properties of SASI materials was thoroughly elucidated. This established a comprehensive research framework spanning from microstructural design to the realization of macroscopic functionality.

**Figure 6:**
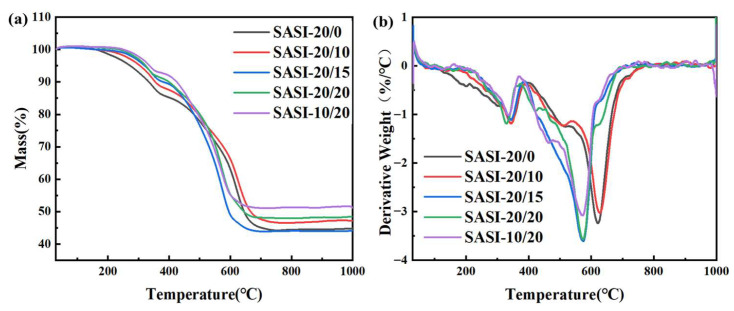
Thermogravimetric analysis curves of five typical SASI composites: (**a**) TG; (**b**) DTG.

As illustrated in Figure 6, the TG and DTG curves of five groups of SASI samples with varying n-MS loading levels in a nitrogen atmosphere are presented. The characteristics of the curves clearly indicate that the thermal decomposition process of SASI composites in a nitrogen atmosphere occurs in two distinct stages. The initial stage occurs within the temperature range of 200–380 °C. The mass loss during this stage is primarily attributed to the volatilisation of small-molecule compounds, such as propanol, which are generated during the curing of the SASI samples, and the desorption of adsorbed water from the material surface. The second stage is concentrated within the temperature range of 400–780 °C and is presumed to originate primarily from the thermal degradation of the silicone rubber matrix, accompanied by the thermal decomposition of n-MS.

It is evident that as the proportion of n-MS increases, three changes can be observed. Firstly, weight loss prior to 400 °C is significantly reduced and slowed; this may be because the increased n-MS reduces the residual small-molecule compounds, allowing those generated during the spraying stage to be more effectively removed from the system. Consequently, it can be posited that following the incorporation of n-MS, particularly prior to 300 °C, the thermal stability of the silicone rubber system is significantly enhanced; moreover, the weight loss process of the material is accelerated to approximately 500 °C. According to the relevant literature, when the temperature of a magnesium silicate aqueous solution exceeds 430 °C, reactions of dehydroxylation occur between the Mg-OH and Si-OH groups in the structure [35]. It is evident that the proportion of n-MS in the SASI material has a significant impact on the thermal decomposition curve of n-MS, as evidenced by the TG/DTG curves. The primary function of flame-retardant fillers in silicone rubber insulating materials is through endothermic decomposition, moisture release and the formation of oxide residues, thereby suppressing thermal runaway [36]. In the case of n-MS-reinforced SASI systems, the moisture released during thermal decomposition can lower the material’s surface temperature, whilst the oxide residues formed by endothermic decomposition act as carbonisation templates to promote the carbonisation reaction of the silicone rubber matrix, thereby reducing mass loss during thermal decomposition and increasing the char yield. Thirdly, as the n-MS loading increases, the char yield of the SASI material exhibits a gradual upward trend, with the SASI-10/20 sample exhibiting the highest char yield. This outcome is consistent with the established flame-retardant mechanism of n-MS; while there is a marginal reduction in the material’s thermal decomposition temperature, there is a substantial enhancement in its flame-retardant performance through the promotion of charring and the suppression of thermal feedback. The TG test in this article was conducted under an air atmosphere and slow heating conditions, which can only indirectly reflect the material’s thermal decomposition and carbonization trend and cannot fully simulate the high-temperature and high-speed erosion environment of real ablation.

In summary, the incorporation of n-MS has been shown to have a positive role in enhancing the flame-retardant properties of the SASI material. The thermal decomposition behavior of the material influences not only its thermal stability but may also further modulate the microstructure of ablation products during high-temperature ablation. In this study, five groups of SASI samples with different n-MS loading levels were ablated for 20 s using an oxy-acetylene flame at approximately 3000 °C. Subsequent to the process of ablation, the microstructure of the ablated layer surface was systematically characterized and analyzed in depth for each sample. As illustrated in Figure 6, a high-power microscope was utilized to observe the microstructural features of the ablated layer surface for five representative samples. In conjunction with the thermal decomposition patterns shown in Figure 6, a detailed analysis is provided below.

**Figure 7:**
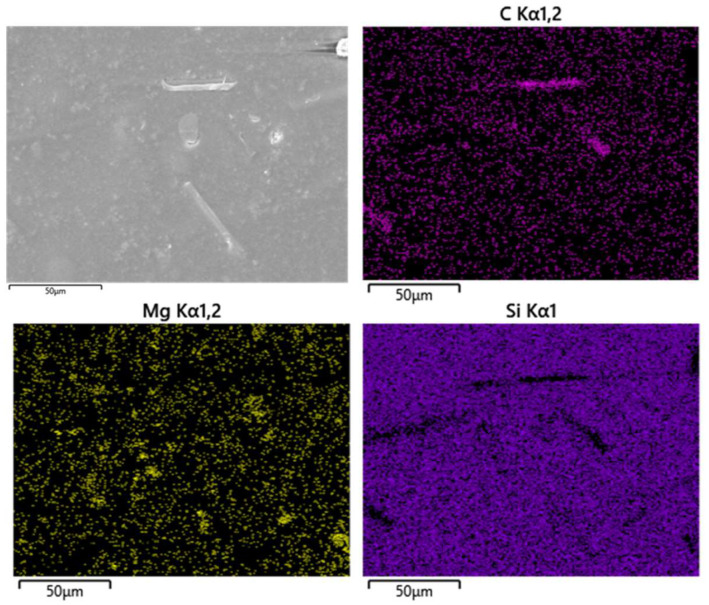
SEM-EDS mappings of the cured SASI-20/15 sample surface.

As shown in the cross-sectional SEM image of SASI-20/15 (Figure 7), the nanoflower fillers are largely uniformly dispersed in the silicone matrix. No prominent filler debonding or pull-out is observed on the fracture surface, and no significant gaps exist between the silicone matrix and the hierarchical nanoflower petals. These observations suggest that the silicone matrix forms relatively tight encapsulation and deep infiltration around the nanoflower fillers. Meanwhile, the crack propagation path tends to be deflected owing to the mechanical blocking and interfacial anchoring effects of the fillers. Furthermore, EDS elemental mapping and line scan analyses reveal that Si and the characteristic elements of the nanoflowers are continuously distributed across the interface without distinct phase separation, supporting that the silicone matrix has fully penetrated into the interlayer voids between nanoflower petals. Collectively, these results demonstrate from both morphological and compositional perspectives that a robust interfacial interlocking structure can be formed between the silicone matrix and nanoflower fillers. Such mechanical interlocking helps to effectively improve the interfacial adhesion and fracture toughness of the composite.

**Figure 8:**
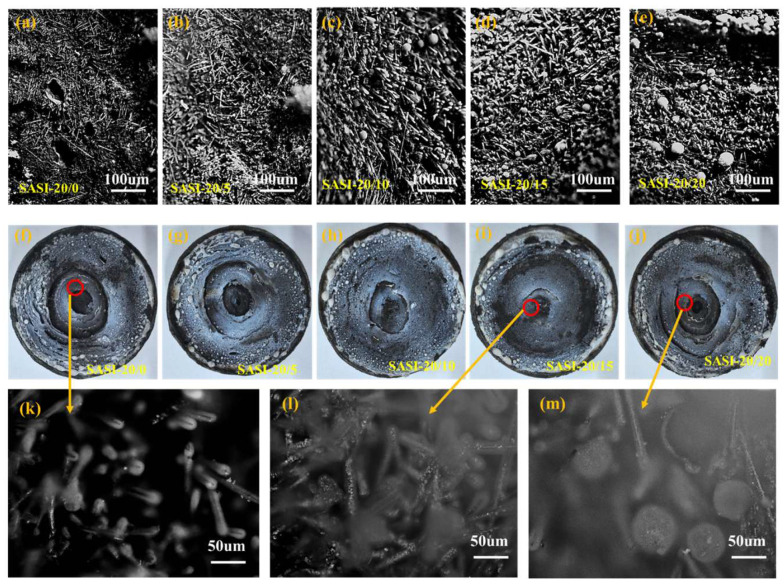
(**a**–**e**,**k**–**m**) Micro-morphologies of the ablation layer surfaces of five typical samples under a high-power microscope; (**f**–**j**) appearances of the ablation layers of SASI composites with different n-MS contents.

It can be observed from Figure 8 that with the gradual increase in n-MS addition, the microstructure of the ablation products of SASI samples after high-temperature oxyacetylene ablation displays a noticeable trend of evolution from a disordered and loose state to a denser one, and residual traces of molten droplets can be clearly observed on the fiber surface. A key mechanism underlying this phenomenon is that, in a high-temperature ablation environment, n-MS undergoes thermal decomposition and contributes to the formation of a continuous ceramic protective layer. This ceramic phase then interacts synergistically with molten HFS and the SiO_2_ liquid phase derived from the thermal degradation of silicone rubber. During this process, the resulting composite liquid phase gradually permeates into the carbon fiber bundles and constructs a continuous melt film with appropriate thickness and viscosity on the material surface. This melt film exhibits excellent resistance to gas flow scouring, which helps suppress erosion of the ablation layer by high-temperature gas flow, thus significantly improving the compactness of the ablation layer surface [37,38,39].

It is worth noting that distinct molten droplet spheres appear on the ablation layer surface of the SASI-20/20 sample. It is proposed that this arises when an excessive amount of composite liquid phase condensed inside the fibers escapes to the material surface and is subsequently scoured by the high-speed oxyacetylene flow, eventually leading to discrete droplet-like residues. This abnormal behavior appears to be closely related to the high n-MS loading in the SASI-20/20 sample and the excess liquid phase generated during thermal decomposition. This finding further indicates that the n-MS addition should be controlled within a reasonable range to optimize the overall performance of the material, which aligns well with the analytical conclusions regarding mechanical properties and density evolution discussed above.

## 4. Conclusions

The present study adopted a materials design approach, utilizing a hydrothermal method to synthesize magnesium silicate (n-MS) with a nano-florette structure in a controlled manner, and systematically investigated its regulatory effects on the sprayability, mechanical properties, flame retardancy and ablation resistance of sprayable silicone rubber insulation (SASI). The study indicates that the loading of n-MS is a key design parameter determining the comprehensive performance of SASI composites. When the loading is controlled within 15 parts, the SASI-20/15 coating exhibits a smooth, defect-free surface and demonstrates good spray coating process adaptability. Its Shore A hardness was 78, tensile strength reached 3.70 MPa, and elongation at break was 270%. Meanwhile, linear ablation rate and mass ablation rate were reduced by 41.8% and 67.1%, respectively, achieving a synergistic optimization of mechanical and ablation resistance properties. As the n-MS content increases, the material hardness continues to rise, whilst the tensile strength and elongation at break exhibit a non-monotonic trend of first increasing and then decreasing. This reflects the complex influence of n-MS’s dispersion state within the matrix and its interfacial bonding mode on mechanical behavior. With regard to thermal stability and flame-retardant mechanisms, although n-MS appears to slightly reduce the onset thermal decomposition temperature of SASI, it also helps to enhance flame-retardant performance by facilitating high-temperature charring behavior. Under oxyacetylene ablation conditions, n-MS further transforms to form a continuous and dense ceramicised protective layer, which interacts synergistically with the molten phase and consequently effectively reinforces the ablation resistance of the material. Nevertheless, excessive incorporation of n-MS tends to induce agglomeration defects and result in the deterioration of overall performance. These observations suggest that accurate control of its addition level represents a key factor in achieving favorable material performance. Overall, this study offers a feasible strategy for the preparation of high-performance sprayable thermal protection materials intended for aerospace applications.

## Figures and Tables

**Table 1:** Formulation Table of Nanostructured Magnesium Silicate in the SASI.

Sample	B-HTPS	HFS	MCF	n-MS	TPOS	DBTDL	PE
SASI-20/0	100	20	40	0	8	1.5	100
SASI-20/5	100	20	40	5	8	1.5	100
SASI-20/10	100	20	40	10	8	1.5	100
SASI-20/15	100	20	40	15	8	1.5	100
SASI-20/20	100	20	40	20	8	1.5	100
SASI-15/20	100	15	40	20	8	1.5	100
SASI-10/20	100	10	40	20	8	1.5	100
SASI-5/20	100	5	40	20	8	1.5	100

Note: All data are given in grams.

## Data Availability

The original contributions presented in this study are included in the article. Further inquiries can be directed to the corresponding author.
